# Cytidine and dCMP Deaminases—Current Methods of Activity Analysis

**DOI:** 10.3390/ijms26168045

**Published:** 2025-08-20

**Authors:** Anna Ligasová, Martina Horejšová, Radana Brumarová, David Friedecký, Karel Koberna

**Affiliations:** 1 Institute of Molecular and Translational Medicine, Faculty of Medicine and Dentistry and Czech Advanced Technology and Research Institute, Palacký University Olomouc, Hněvotínská 5, 779 00 Olomouc, Czech Republic; 2 Laboratory of Inherited Metabolic Disorders, Department of Clinical Chemistry, Palacký University and University Hospital Olomouc, Zdravotníků 248/7, 779 00 Olomouc, Czech Republic; martina.horejsova@upol.cz (M.H.); radana.brumarova@upol.cz (R.B.); david.friedecky@fnol.cz (D.F.); 3 Institute of Molecular and Translational Medicine, Faculty of Medicine and Dentistry, Palacký University Olomouc, Hněvotínská 5, 779 00 Olomouc, Czech Republic

**Keywords:** cytidine deaminase, dCMP deaminase, spectrophotometry, cell-based approaches, LC-MS, modified nucleosides

## Abstract

Cytidine deaminase (CDA) and deoxycytidine monophosphate deaminase (DCTD) play crucial roles in pyrimidine metabolism, affecting DNA synthesis, cell cycle progression, and the efficacy of numerous nucleoside analog-based chemotherapeutics. Given their significance, accurate and sensitive measurement of their enzymatic activity is paramount for both fundamental biochemical research and clinical applications. This review provides a comprehensive overview of the methodologies used to assess CDA and DCTD activity, both established and emerging. We systematically categorize and discuss various approaches, including spectrophotometric, fluorimetric, liquid chromatography-based (Ultraviolet-Visible, fluorescence, and mass spectrometry), radiometric, and cell-based assays. For each method, we present its underlying principles, advantages, and limitations. Furthermore, we draw comparisons across the techniques to highlight their suitability for specific research questions.

## 1. Introduction

Cytidine deaminase (CDA) and deoxycytidine monophosphate deaminase (dCMP deaminase or DCTD) play significant roles in pyrimidine metabolism, where they catalyze the deamination of specific cytidine substrates [1]. Reliable and sensitive assessment of enzymatic activities is fundamental for both basic research into nucleotide metabolism and optimizing nucleoside analog-based therapies.

**Figure 1:**
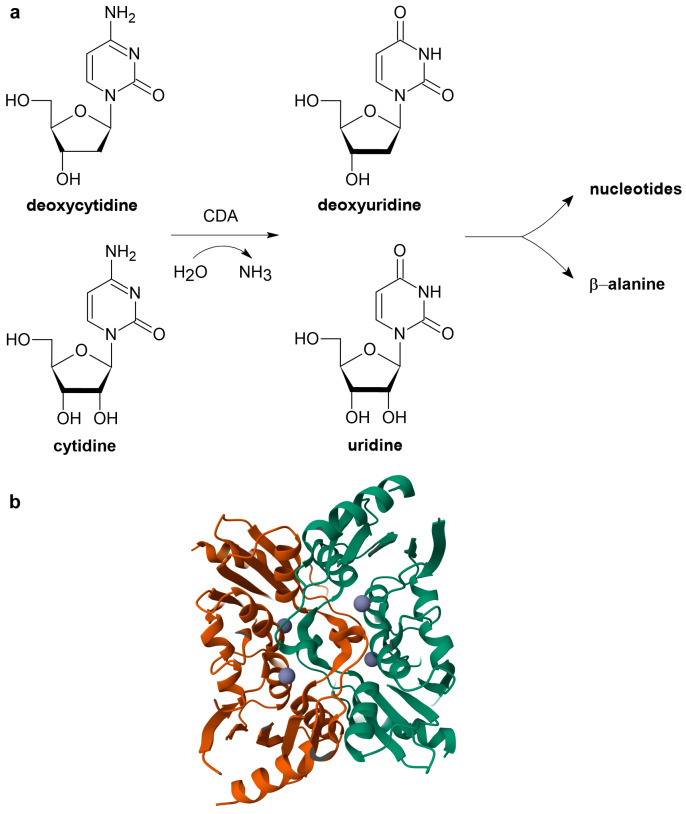
Role of CDA and its structure. (**a**) Simplified scheme of CDA’s role in nucleotide metabolism. (**b**) The tetrameric structure of CDA (PDB: 1MQ0; https://www.rcsb.org/structure/1MQ0, accessed on 5 August 2025), which is formed by two dimers (shown in brown and green colors). Zinc ions are shown as violet spheres.

CDA (EC 3.5.4.5) catalyzes the hydrolytic conversion of cytidine to uridine and deoxycytidine to deoxyuridine (Figure 1) [1,2,3]. This activity is important for the efficient recycling of nucleosides obtained from RNA and DNA degradation, thereby contributing to the maintenance of nucleotide pool homeostasis. Additionally, CDA participates in the pyrimidine catabolic cycle, which serves as a carbon and nitrogen source for the cell and leads to the formation of β-alanine (Figure 1a) [2]. Human CDA is a homotetrameric enzyme composed of four identical subunits, each with a molecular weight of approximately 16 kDa [4] (Figure 1b). The active site of each subunit contains an essential zinc atom [5,6,7,8]. This zinc atom is coordinated into the correct position by three negatively charged cysteine residues (C65, C99, and C102) and plays a crucial role in the catalytic process by activating a water molecule, thus creating a hydroxyl nucleophile essential for initiating the deamination reaction [5,6,7,8]. The glutamic acid residue at position 67 (E67) also plays an important role by initiating catalysis, as its carboxyl group enables the deprotonation and protonation of the water molecule and the substrate’s amino group. This subsequently initiates a nucleophilic attack by the hydroxide on the C4 of the pyrimidine ring [5,9], leading to the formation of a tetrahedral reaction intermediate in which the pyrimidine ring interacts with the hydroxyl group, the carboxylate part of the glutamate, and the zinc ion [2,10].

CDA deficiency can lead to the abnormal accumulation of 2′-deoxycytidine 5′-triphosphate (dCTP), which subsequently significantly slows down the progression of DNA replication forks and results in the presence of under-replicated genomic DNA [11]. Recently, Frances and colleagues demonstrated that CDA expression (even independently of its catalytic activity) can influence mitochondrial metabolites, cellular respiration, ATP production, and mitochondrial biogenesis in pancreatic cancer cells [12].

**Figure 2:**
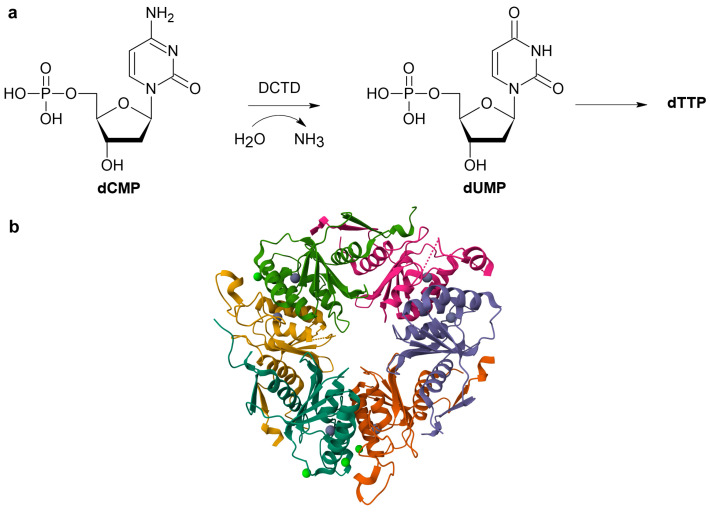
Role of DCTD and its structure. (**a**) Simplified scheme of DCTD’s role in nucleotide metabolism. (**b**) The hexameric structure of DCTD (PDB: 2W4L; https://www.rcsb.org/structure/2W4L, accessed on 5 August 2025). The catalytic zinc ions are shown as violet spheres. Chlorine ions are shown as green spheres.

DCTD (EC 3.5.4.12) catalyzes the deamination of deoxycytidine-5′-monophosphate (dCMP) to deoxyuridine-5′-monophosphate (dUMP) [13]. This reaction directly links DCTD to the de novo thymidine 5′-triphosphate (dTTP) synthesis pathway, as dUMP serves as a direct substrate for thymidylate synthase (TS). TS synthesizes dTMP from dUMP, which is then phosphorylated to dTTP [14]. Thus, DCTD ensures a sufficient supply of dTTP for DNA replication and repair (Figure 2a) [15]. In addition to its primary role, DCTD is also involved in the deamination of epigenetically modified 5-hydroxymethyl-dCMP (hmdCMP) to potentially cytotoxic 5-hydroxymethyl-dUMP (hmdUMP) [13,14,16]. DCTD activity is allosterically regulated by the ratio of dCTP (activator) and dTTP (inhibitor) [13,16,17]. This regulatory mechanism ensures a balanced production of deoxynucleotides within the cell. Any dysfunction or dysregulation of DCTD directly leads to unbalanced dNTP (deoxynucleotide triphosphate) pools, subsequently increasing the mutation rate and inducing replication stress [16,18,19].

In eukaryotes, DCTD is a hexamer [16] (Figure 2b). Each of its six protomers, approximately 20 kDa in size, contains one catalytic site and one allosteric site, which regulates the enzyme’s activity. As with CDA, zinc is required for the DCTD active site to function [17]. The catalytic site contains conserved HxE and PCxxC motifs, which are characteristic of this enzyme family and are involved in zinc binding and in the catalysis itself. The zinc ion is tetrahedrally coordinated by two cysteine residues, one histidine residue, and a water molecule. The glutamate residue is essential for catalysis and serves as a proton transporter [10,17].

**Figure 3:**
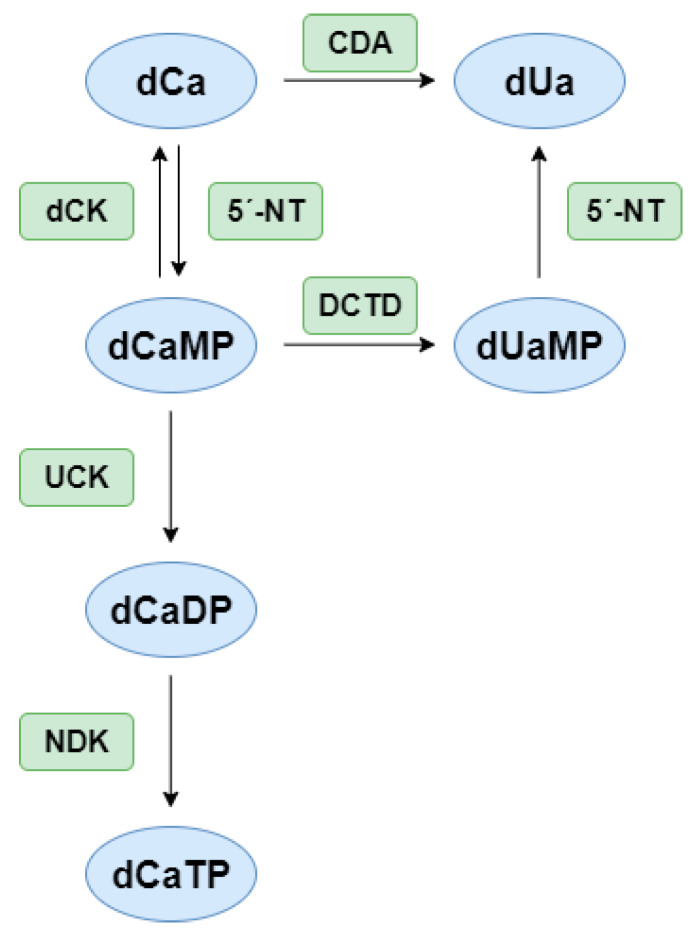
Simplified scheme of the activation and inactivation pathways of cytidine analogs (dCas). dUa—2′-deoxyuridine analog; dCaMP, dCaDP, dCaTP—mono-, di-, triphosphate form of dCas; dUaMP—monophosphate form of dUa; dCK—deoxycytidine kinase; UCK—UMP-CMP kinase; NDK—nucleoside diphosphate kinase; CDA—cytidine deaminase; DCTD—dCMP deaminase; and 5′-NT—5′-nucleotidases. Adapted from [21].

The clinical significance of both deaminases is substantial. In oncology, these enzymes play an important role in the metabolism of cytidine analogs, a widely used group of chemotherapeutic agents for treating various types of cancers, including myelodysplastic syndromes (MDS), acute myeloid leukemia (AML), pancreatic carcinoma, and lung cancer [20]. Figure 3 shows a simplified diagram of cytidine analog metabolic pathways and the involvement of CDA and DCTD.

High expression and/or activity of CDA and DCTD can result in the rapid deamination and subsequent inactivation of nucleoside analogs. This reduces both their systemic exposure and therapeutic efficacy in cancer cells. Conversely, decreased CDA/DCTD activity or their pharmacological inhibition can result in increased exposure to the active form of the drug, potentially leading to more severe side effects (e.g., increased toxicity). Substances typically used in the treatment of malignancies include cytarabine (1-β-D-arabinofuranosylcytosine, ara-C), gemcitabine, and decitabine [22,23,24,25,26,27]. The application and effectiveness of these substances are also considerably influenced by transport, phosphorylation, and mutual competition with natural nucleosides [28,29].

For some drugs, CDA actually plays an activating role. Capecitabine, for instance, is initially converted to 5′-deoxy-5-fluorocytidine by the enzyme carboxylesterase. Next, CDA deaminates this compound to form 5′-deoxy-5-fluorouridine [1,30,31]. This compound is then further metabolized in subsequent steps to 5-fluoro-2′-deoxyuridine monophosphate (FdUMP), which inhibits the enzyme TS. This inhibition disrupts the synthesis of dTTP and ultimately leads to the inhibition of DNA replication [32,33,34,35].

In addition, in the presence of mutagens like 5-hydroxymethyl-2′-deoxycytidine (5hmdC) and 5-formyl-2′-deoxycytidine (5fdC), their deamination produces uridine derivatives (5hmdU and 5fdU). These derivatives can subsequently be incorporated into genomic DNA, leading to extensive DNA damage, arrest of DNA synthesis and the cell cycle, and, ultimately, cell death [36,37].

The expression and activity of CDA and DCTD, and consequently treatment response and sensitivity to cytidine analogs/drugs, vary significantly between individuals due to genetic polymorphisms in the genes encoding these enzymes. These polymorphisms can alter enzymatic activity and drug pharmacokinetics. A wide range of genetic variations, including single nucleotide polymorphisms (SNPs), have been identified in the human *CDA* and *DCTD* genes. These SNPs can significantly affect enzymatic activity, protein expression, and subsequently impacting the pharmacokinetics and pharmacodynamics of nucleoside analogs [38]. Well-characterized and clinically relevant nonsynonymous SNPs in the *CDA* gene include A79C (Lys27Gln) [39] and G208A (Ala70Thr) [40]. In the *DCTD* gene, the nonsynonymous coding SNP A172G (Asn58Asp) leads to a loss of activity [23]. Due to these SNPs, a significant portion of the patient population exhibits altered drug metabolism. It can result in a spectrum of responses ranging from hypersensitivity to complete resistance.

Reliable and sensitive methods for measuring the enzymatic activity of CDA and DCTD are essential to fully understand their biological functions and regulation. The basic strategy for determining CDA and DCTD activity relies on monitoring the depletion of substrates or the accumulation of products. This review comprehensively examines current and established methods for analyzing CDA and DCTD enzyme activity, focusing on their practical aspects, advantages, and limitations. The paper covers the principles and applications of spectroscopic techniques, including traditional Ultraviolet-Visible (UV-Vis) spectrophotometry and modern fluorogenic assays. It also includes chromatographic approaches, methods utilizing mass spectrometry or their combinations, and techniques suitable for studying activity in cellular systems.

**Figure 4:**
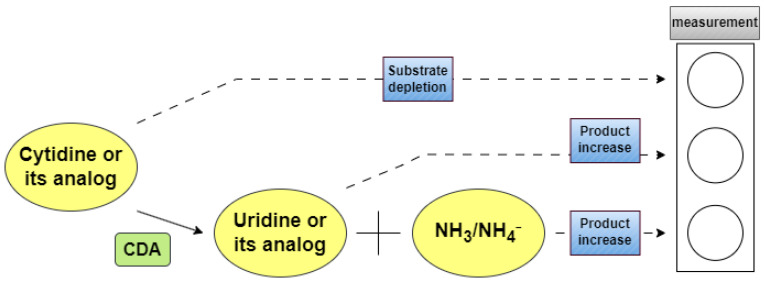
Scheme of the basic principle of CDA activity measurement.

When determining CDA activity, analytical strategies most commonly involve tracking the decrease in cytidine substrates or quantifying the reaction products, namely uridine (or deoxyuridine) and ammonia (Figure 4). Similarly, for DCTD, activity is typically assessed based on the formation of dUMP or ammonia, or the depletion of dCMP.

## 2. Spectrophotometric and Fluorimetric Assays

These methods are based on either measuring light absorption (spectrophotometry) or its emission (fluorimetry).

The principle of spectrophotometric methods is to measure the changes in absorbance in the ultraviolet (UV) or visible (Vis) regions of the spectrum that accompany the enzymatic conversion of a substrate to a product. According to the Beer–Lambert law, this change is directly proportional to the change in the concentration of the substances involved [41].

In contrast, fluorimetric methods (fluorescence spectroscopy) are based on fluorescence, where specific molecules (fluorophores), after absorbing light (excitation) at a particular wavelength, emit light at a longer wavelength (emission). The difference between the excitation and emission wavelengths is called the Stokes shift. The intensity of the emitted fluorescence is directly proportional to the concentration of the fluorescing substance. Fluorimetric methods are generally known for their high sensitivity, often exceeding that of absorption spectrophotometry, and allow for the detection of very low analyte concentrations [42].

### 2.1. Methods Based on Absorption Spectrophotometry

For the determination of CDA and DCTD activity, techniques based on the measurement of light absorption are among the oldest and most widely used. These methods can be further categorized into direct methods, which monitor changes in the UV-Vis absorption of substrates or products, and indirect methods, which detect one of the deamination reaction products, typically ammonia, using a subsequent colorimetric reaction or enzyme coupling.

#### 2.1.1. Direct UV-Vis Spectrophotometric Assays

In the case of direct methods, enzyme activity is measured by monitoring the change in absorbance at a specific wavelength. This change is a direct result of the conversion of a substrate (e.g., cytidine/deoxycytidine) to a product (e.g., uridine/deoxyuridine), where the substrate and product exhibit different absorbance at that particular wavelength [43,44]. For example, Cohen & Wolfenden (1971, Figure 5) or Vita et al. (1985) utilized this approach to measure CDA activity by analyzing the decrease in absorbance at 282 nm as cytidine was converted to uridine, using CDA purified from *E. coli* [43,44].

While uridine is the product of cytidine deamination, its direct spectrophotometric quantification for CDA activity determination is not commonly used. However, uridine quantification as a product is utilized, especially with liquid chromatography (LC) coupled with tandem mass spectrometry (MS).

The advantages of direct spectrophotometric methods include the ability to continuously monitor the enzymatic conversion of cytidine to uridine in real time, which is useful for determining initial reaction rates. The technique is non-destructive to the analyzed solution in the cuvette, theoretically allowing for further analyses. Finally, the instrumentation—a standard UV-Vis spectrophotometer—is readily available, and both measurement and basic data processing are relatively straightforward. The main disadvantage is the low selectivity of the method, as other substances in the sample that absorb at the same wavelength can interfere with the measurement. This limitation is highlighted by the fact that the foundational studies employing this method, such as those by Cohen & Wolfenden (1971) [43] and Vita et al. (1985) [44], were conducted using highly purified enzyme preparations. This experimental choice underscores that for complex biological mixtures, such as cell lysates, interference would be a significant issue requiring sample purification. Measurement accuracy can also be affected by the physical properties of the sample, such as light scattering in the presence of undissolved particles. Applying this method to substrates other than cytidine requires careful selection of a wavelength where the change in absorbance is maximal and potential interferences are minimal [45].

#### 2.1.2. Indirect Spectrophotometric Assays

The indirect determination of CDA/DCTD activity is based on release of ammonia during the deamination of cytidine substrates (cytidine, deoxycytidine, and dCMP) [2]. This ammonia can then be detected using various colorimetric methods or coupled enzymatic reactions that lead to a change in absorbance.

As early as 1969, Newton Ressler described a method for determining DCTD activity in human tissues and serum, which utilized the colorimetric detection of ammonia via the Berthelot reaction. In this assay, the liberated ammonia reacts with phenol and hypochlorite in an alkaline environment to form a blue-green indophenol dye. The absorbance of this dye is then measured at 660 nm. Ressler used an incubation time of 60 min at 37 °C for tissue homogenates or 18 h at 22 °C for serum. The method was described as relatively reproducible, sensitive, and simple to perform. It is approximately ten times more sensitive than the common Nessler’s reaction, which relies on ammonia reacting with an alkaline solution of mercuric potassium iodide (Nessler’s reagent) to produce a yellow-brown to red-brown color or precipitate, with intensity proportional to ammonia concentration [46].

Ressler’s method has been modified several times. For example, Williams and Jones (1975) used a modified reaction to analyze the produced ammonia for DCTD determination in pregnancy serum. This modification utilized the catalytic effect of sodium nitroprusside on the reaction of salicylate with hypochlorite, producing a green color in the presence of ammonia. The reaction was carried out for 18 h at 22 °C, and absorbance was measured at 560 nm [47]. One advantage of this modification was that sample deproteinization was not required prior to ammonia detection. However, the long 18 h incubation period required for ammonia production and the lower incubation temperature (22 °C) were drawbacks [48].

**Figure 6:**
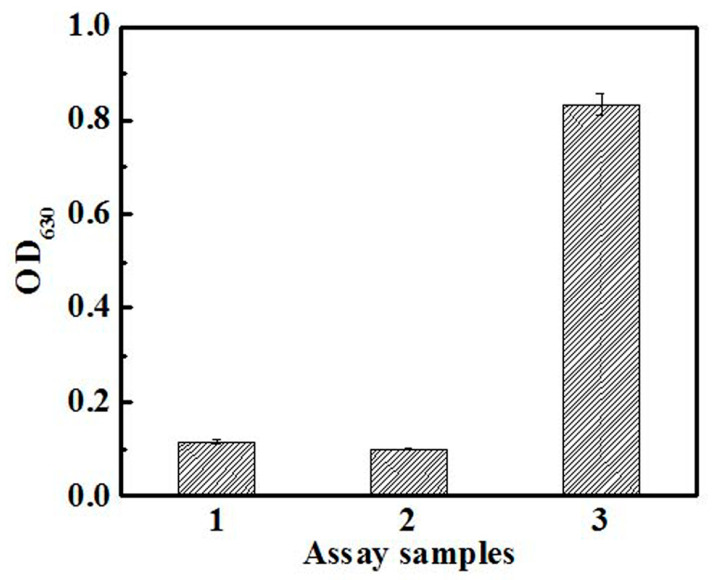
An example of the determination of CDA activity using indirect spectrophotometric assay. On the graph, CDA activity was measured in these samples: 1—reaction reagent without CDA and cytidine; 2—reaction reagent with CDA and without cytidine; 3—reaction reagent with CDA and cytidine. The concentration of cytidine was 1 mmol/L. The experiments were performed in triplicate. Adapted from [49].

Dong et al. (2015) utilized a method for CDA activity determination based on the enzymatic conversion of cytidine to uridine and ammonia, followed by colorimetric quantification of the released ammonia using the indophenol method (Figure 6). The enzymatic reaction for ammonia production was carried out for 20 min at 37 °C, followed by 30 min at 37 °C for color development, after which absorbance was measured at 630 nm. The sensitivity of the method, assessed by the dependence of signal on cytidine concentration, varied depending on the medium used: the linear detection range in water was 0.05–10 mM, in M9 (M9 minimal salts) medium 0.058–10 mM, while in complex LB (the Lysogeny Broth) medium, the range was 2.5–10 mM. When using 10× diluted LB medium, the linear range was 0.102–10 mM [49].

Targett-Adams and colleagues (1975) described a method for determining DCTD activity in serum, where the liberated ammonia was specifically quantified using a coupled enzymatic reaction with glutamate dehydrogenase (GLDH). This enzyme catalyzes the amination of α-ketoglutarate by ammonia, coupled with the oxidation of nicotinamide adenine dinucleotide (NADH) to NAD^+^, which leads to a measurable decrease in absorbance at 340 nm. Serum incubation with dCMP proceeded for 4 h at 37 °C. Subsequently, after adding α-ketoglutarate and NADH and a 15 min pre-incubation at 22 °C to allow endogenous reactions (i.e., reactions consuming NADH independently of ammonia from dCMP deamination) to subside, GLDH was added. After another 15 min, the decrease in absorbance was measured. Advantages of this modification included its sensitivity, a shorter incubation time sufficient for ammonia production, and incubation at a higher temperature, resulting in a threefold increase in ammonia yield [48].

Indirect methods based on ammonia detection generally offer several advantages. For example, they can be very sensitive, as demonstrated by Ressler (1969) in comparing the Berthelot reaction with Nessler’s, and by Dong et al. (2015) and Targett-Adams et al. (1975) using the indophenol and enzymatic GLDH methods, respectively [46,48,49]. Allowing for longer incubation to accumulate ammonia can further increase sensitivity for samples with low enzyme activity [46]. Some modifications, like that by Williams and Jones (1975), eliminate the need for sample deproteinization [47]. The method by Targett-Adams et al. (1975) significantly reduced the total analysis time to less than 4.5 h by optimizing temperature and using sensitive enzymatic ammonia detection [48]. Furthermore, Dong et al. (2015) adapted their method for high-throughput screening in 96-well plates [49].

However, these methods have disadvantages, notably the potential for interference from ammonia that can arise in biological systems through numerous other metabolic pathways (e.g., amino acid deamination, purine catabolism, or activity of other amidohydrolases). This can lead to a high background, masking the specific ammonia production by the enzyme being measured. As Dong et al. (2015) [49] and Targett-Adams et al. (1975) [48] point out, it is, therefore, absolutely essential to use carefully designed controls that contain biological material but no specific substrate for CDA/DCTD. This accounts for these non-specific sources of ammonia. Further interference can be caused by components of complex media containing amino groups, which affect the indophenol reaction. For example, Dong et al. (2015) [49] systematically demonstrated that while simple carbon sources like glucose had no effect, complex nutritional components such as yeast extract and tryptone substantially impacted the accuracy of their indophenol-based assay, likely due to the presence of amino groups that directly react with the detection reagents. Some protocols may also require long incubation times (e.g., 18 h in Ressler’s (1969) method [46] for serum or Williams and Jones’ (1975) method [47]). Coupled enzyme systems, such as the GLDH method, require optimal conditions for all involved enzymes and minimal interference during NADH absorbance measurements. One way to achieve this is the pre-incubation to allow endogenous reactions to subside, as described by Targett-Adams et al. (1975) [48].

### 2.2. Fluorescence-Based Methods

#### 2.2.1. Indirect Fluorimetric Assays

**Figure 7:**
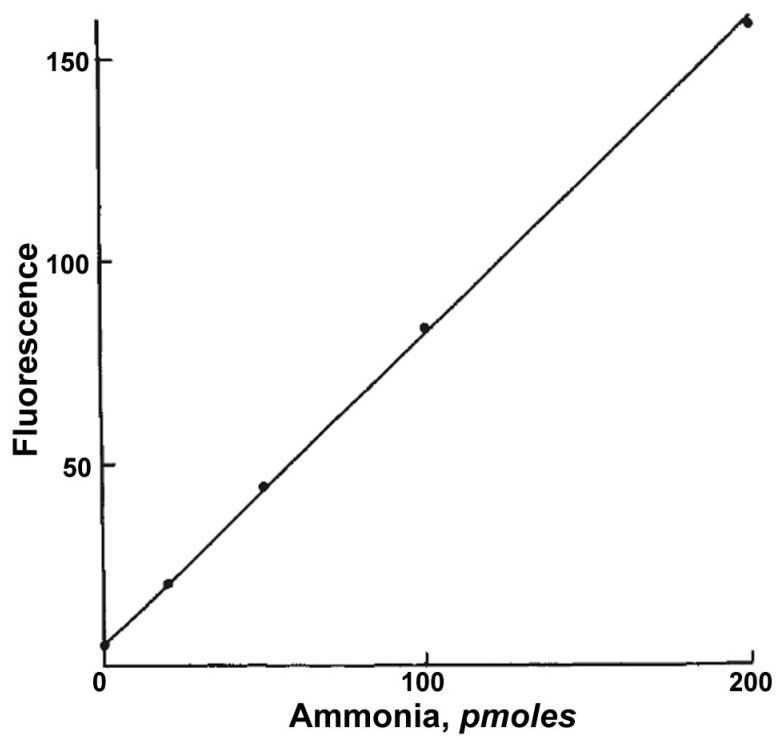
An example of measurement of CDA activity using indirect fluorimetric assay. Standard curve for ammonia assay performed in a final volume of 2 μL on samples of 1 nL. Fluorescent droplets were taken into individual capillaries (see text in the citation). Fluorescence, measured 45 min after mixing sample and reagent, is expressed in arbitrary units. Each point is the mean of duplicate determinations. Fluorescence at zero ammonia is that of the reagent blank. The line is the least squares linear regression line. Adapted from [50].

Beyond colorimetric absorption-based methods, ammonia released by deamination reactions can also be quantified using highly sensitive fluorescence methods. These methods typically rely on ammonia reacting with a fluorogenic reagent, such as o-phthaldialdehyde (OPA), in the presence of a thiol compound, producing an intensely fluorescent product [17,50,51]. Mroz et al. (1982) described a modification of such a method, where they used OPA with thioglycolic acid (instead of mercaptoethanol) at pH 7.4, resulting in improved specificity for ammonia. Fluorescence measurement was taken at least 45 min after mixing the reagents, with excitation wavelengths in the 400–440 nm range and emission measured above 455–475 nm. This method enabled the determination of picomolar to subpicomolar amounts of ammonia (Figure 7) [50].

As with colorimetric ammonia detection methods, it is crucial here to account for potential interference caused by endogenous ammonia or its production via other metabolic pathways within the biological sample, as was highlighted for the analogous spectrophotometric assays by Targett-Adams et al. (1975) [48] and Dong et al. (2015) [49].

The principle of fluorescent ammonia detection is well-established. In fact, some commercially available kits designed for CDA activity determination (e.g., from Assay Genie, Ireland or Abcam, United Kingdom) were based on this principle. These kits typically provided optimized reagents for the sensitive fluorescent detection of ammonia released from cytidine by CDA.

#### 2.2.2. Direct Fluorimetric Assays

Direct fluorogenic substrates are engineered to undergo a change in their fluorescence properties as a direct result of CDA’s enzymatic activity. This allows for continuous, real-time monitoring of the reaction.

A significant advancement in this field was the development of isomorphic fluorescent nucleosides by Yitzhak Tor’s group [52]. These synthetic analogs are designed to minimally disrupt the natural structure and function of nucleosides while exhibiting useful photophysical properties, such as emission in the visible spectrum. Their function is based on the principle that the enzymatic deamination of a fluorescent cytidine analog to its corresponding uridine analog results in a change in fluorescence—either a change in emission intensity, a shift in the emission maximum, or a combination of both [53,54].

**Figure 8:**
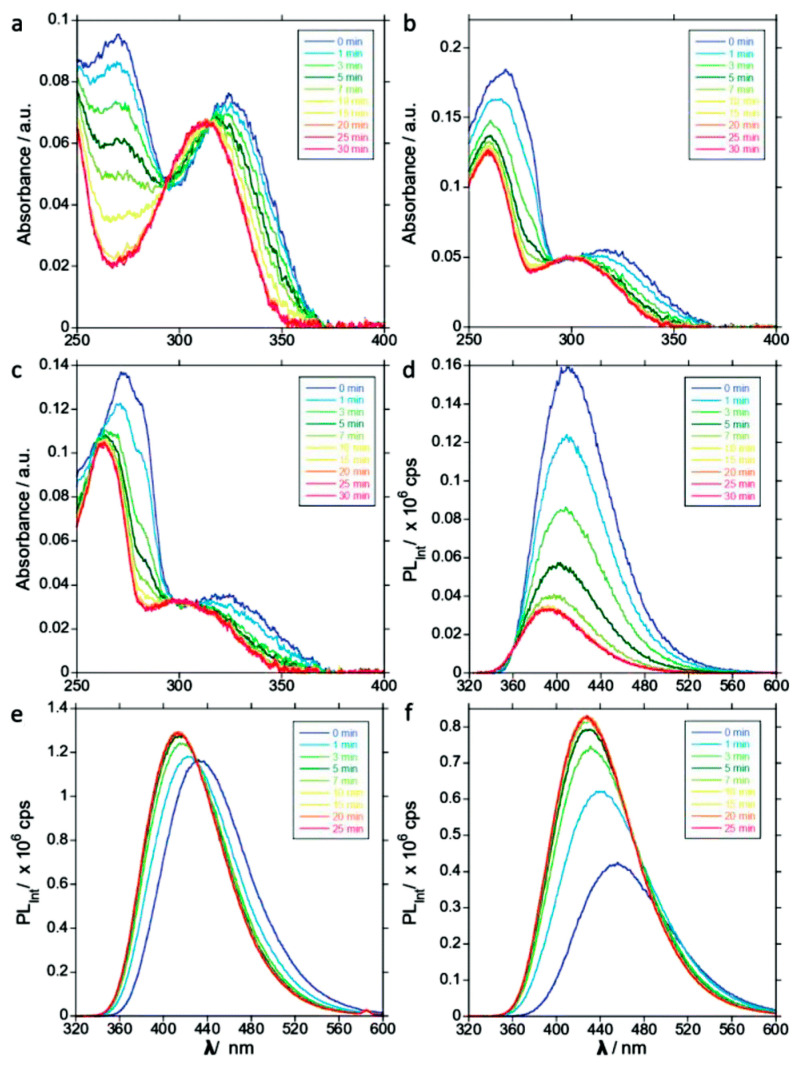
An example of the measurement of CDA activity using direct fluorimetric assay. Steady state absorption traces of (**a**) tzC, (**b**) thC, and (**c**) mthC CDA-mediated conversion to tzU, thU, and mthU, respectively, over a time range from 0 to 30 min; steady state emission traces of (**d**) tzC, (**e**) thC, and (**f**) mthC CDA-mediated conversion to tzU, thU, and mthU, respectively, over a time range from 0 to 30 min. For clarity, the curves show a time course (0, 1, 3, 5, 7, 10, 15, 20, 25, and 30 min), progressing from the initial state at 0 min (blue) to the final state at 30 min (dark red/purple). The final time point in panels (**e**,**f**) is 25 min. Adapted from [53].

Ludford et al. (2021) thoroughly characterized three such analogs as substrates for CDA: an isothiazolo [4,3-d]pyrimidine analog of cytidine (tzC), a thieno[3,4-d]pyrimidine analog of cytidine (thC), and a methylthieno[3,4-d]pyrimidine analog of cytidine (mthC; Figure 8) [53]. Upon deamination of these analogs, the authors observed the following changes: for the tzC, there was a decrease in emission intensity and a blue shift in its emission. In the case of the thC, the authors observed a slight increase in emission intensity and also a subtle blue shift. Conversely, for the mthC, there was a notable increase in emission intensity and a blue shift [53].

A significant advantage of these analogs, especially the mthC, is their relatively large Stokes shifts (e.g., 8930 cm^−1^ for the aforementioned methylthieno analog). Kinetic parameters, based on Michaelis-Menten kinetics, showed that all three tested analogs are substrates for CDA. Notably, the thC and mthC analogs were deaminated more rapidly than native cytidine. A detailed overview of these substrate properties is available in the work by Ludford et al. (2021) [53]. On the other hand, these methods also have their drawbacks. One major disadvantage is the need to synthesize specific fluorogenic substrates, which can be complex and costly compared to commercially available reagents for traditional spectrophotometric assays. Although designed to be isomorphic, these are still analogs, and their kinetic parameters may differ from native substrates, a factor that requires careful consideration when interpreting data. For instance, the study by Ludford et al. (2021) [53] reported that while the developed analogs were effective substrates, their catalytic efficiency was significantly different from that of native cytidine, with some analogs being deaminated more rapidly. This finding highlights that while these probes are excellent for monitoring enzymatic activity, the kinetic data obtained with them may not be directly equivalent to that of the physiological substrate and requires careful interpretation.

### 2.3. Summary and Comparison of Spectrophotometric and Fluorimetric Approaches

Several factors need to be considered when choosing a method to determine CDA or DCTD activity.

Direct UV-Vis spectrophotometric methods are quick and straightforward for data collection. However, they require optically clear samples and a sufficient difference in molar absorptivity between the substrate and product. They can also be less sensitive for samples with low enzyme activity.

Indirect spectrophotometric methods, which rely on detecting ammonia (either colorimetrically or via enzyme-coupled assays), can offer higher sensitivity. This is particularly advantageous as it allows for the accumulation of the product over longer incubation periods [46] or by using sensitive color reactions [49] and coupled enzyme systems [48]. However, these methods can be more time-consuming and are significantly impacted by interference from endogenous ammonia or its production by other metabolic pathways. This necessitates rigorous use of blank controls.

Fluorimetric methods generally offer the advantage of high sensitivity. Indirect fluorimetric assays that detect ammonia can achieve picomolar detection limits (as suggested by [50]), making them suitable for samples with very low activity, though their dynamic range might be limited for some applications. Direct fluorogenic substrates, like isomorphic nucleosides, enable continuous, real-time monitoring of the reaction with high sensitivity and are particularly well-suited for kinetic studies and inhibitor screening. Their red-shifted spectra can also minimize background interference. However, a general drawback of fluorescence methods can be their sensitivity to fluorescence quenching, sample autofluorescence, and, for specialized probes, their cost, availability, or the complexity of their synthesis.

## 3. Liquid Chromatography and LC-Coupled Detection Methods

LC, including High-Performance Liquid Chromatography (HPLC), is a widely used separation method for both qualitative and quantitative analysis. While LC is a general term for chromatographic techniques using a liquid mobile phase, HPLC is a more advanced form that utilizes high pressure to force the mobile phase through columns packed with smaller particles, leading to superior efficiency, resolution, and faster analysis times compared to traditional LC. Therefore, in the context of modern analytical chemistry and this review, the term LC is often used synonymously with HPLC, representing the high-performance variant of liquid chromatography relevant to the analysis of CDA and DCTD activity.

LC enables the separation of analytes in a mixture based on their different interactions with two phases—a mobile phase and a stationary phase [55,56]. The mobile phase is the liquid that carries the analytes through the chromatographic column, while the stationary phase consists of a solid support on whose surface the functional groups that determine its chemical properties are covalently bonded. The principle of separation is based on the different affinities of the individual components of the sample for the stationary phase. Substances that bind more weakly to the stationary phase travel faster through the column and, therefore, have a shorter retention time. Those with a higher affinity are retained in the column for longer. This results in an efficient partitioning of the components, which can then be detected using analytical techniques such as UV/Vis, fluorescence, or MS.

### 3.1. Separation Modes for CDA/DCTD Substrates and Products

Effective separation of the substrates (cytidine, deoxycytidine, and dCMP) of CDA and DCTD from their uridine products (uridine, deoxyuridine, and dUMP) and their therapeutic analogs is paramount for the study of pyrimidine metabolism and the enzymatic activities of these enzymes. These molecules often share structural similarities and are highly polar, posing specific challenges for separation. LC addresses these challenges primarily through three common separation modes: reversed-phase liquid chromatography (RP-LC), normal-phase liquid chromatography (NP-LC), and hydrophilic interaction liquid chromatography (HILIC) [57].

RP-LC is used to separate semi-polar compounds such as phenolic acids, glycosylated steroids, and alkaloids, among others, most commonly using C8, C18, or phenyl stationary phase columns. Ranjbarian and colleagues also employed RP-LC for nucleotide analysis [57,58]. This technique uses a non-polar stationary phase in combination with polar aqueous-organic mobile phases. Retention increases with the hydrophobicity of the analytes, the surface area of the stationary phase, and the polarity of the mobile phase [59]. In contrast to RP-LC, NP-LC employs a polar stationary phase and a less polar mobile phase, resulting in an elution order that depends on analyte polarity—hydrophobic compounds elute first, followed by more polar compounds [60]. Highly polar compounds that cannot be analyzed by NP-LC due to irreversible adsorption and poor solubility in non-polar or weakly polar solvents can be analyzed using HILIC [61,62]. Similarly to NP-LC, HILIC uses a polar stationary phase, allowing the retention of polar analytes such as carbohydrates, amino acids, vitamins, nucleotides, and related metabolites [57]. However, unlike NP-LC, HILIC permits the use of aqueous solvents, making this separation method fully compatible with electrospray ionization mass spectrometry (ESI-MS). In contrast to RP-LC, gradient elution in HILIC typically begins with a low-polarity organic solvent, and polar analytes are eluted by increasing the proportion of aqueous polar solvents in the mobile phase [63]. The HILIC mode often employs columns such as amide, aminopropyl, or zwitterionic columns.

#### Column Selection

Accurate determination of CDA and DCTD enzyme activity requires the effective separation and quantification of their substrates and products—such as cytidine, deoxycytidine, uridine, and deoxyuridine, as well as their phosphorylated forms like dCMP and dUMP. These molecules often exist at varying concentrations and share similar chemical structures, posing significant analytical challenges for their isolation prior to detection, whether analyzing them in complex biological matrices like cell lysates or purified enzyme preparations, or considering their fate within intact cellular systems. Therefore, the selection of an appropriate chromatographic column is paramount to achieving the necessary resolution and sensitivity for reliable deaminase activity assessment.

As early as 1995, Di Pierro and colleagues developed a robust method involving ion-pairing HPLC on a C18 column, in which a suitable ion-pairing reagent such as tetrabutylammonium hydroxide is used to retain highly polar nucleotides [64]. This approach allows for the simultaneous quantification of mono-, di-, and triphosphate forms of nucleosides and their deoxy analogs, including dCMP and dUMP, without the need for chemical derivatization. It minimizes sample loss and artifacts associated with chemical treatment, and provides high resolution under a controlled pH and organic gradient. To ensure baseline stability, greater reproducibility, and reduced noise, an isocratic HPLC method was developed by Ranjbarian et al., 2022 [58], using a reversed-phase C18 column, tetrabutylammonium bromide as the ion-pairing agent, and acetonitrile as the organic solvent. This method enables simultaneous quantification of dNTPs, rNTPs, and ADP in a single run, offering internal quality control for extraction efficiency. An optional DNA polymerase-based assay step can confirm the identity of dNTP peaks and exclude interfering compounds that co-elute.

Using a porous graphitic carbon column (PGC) allows the separation of structurally similar compounds like dCMP and CMP, which are otherwise difficult to resolve on reversed-phase columns. The graphitic carbon surface behaves like a Lewis base and acts as such towards polar solutes. As the charge of the polar analyte approaches the graphite surface, a charge-induced dipole is formed by the graphite surface, which increases the attraction between the analyte and the carrier. In addition, PGC has the advantage of tolerating a large pH range (0–14) [65,66]. Gradient elution with water/acetonitrile and formic acid provides sufficient resolution and sensitivity for quantitative purposes. However, despite the effectiveness of PGC columns, challenges persisted, particularly relating to analysis time, MS contamination and limitations in resolving certain structural isomers, even with the careful inclusion of ion-pairing agents such as hexylamine and diethylamine to facilitate the elution of polar triphosphates. This led to increasing interest in HILIC as a more MS-compatible and polarity-focused alternative. HILIC significantly improves the retention of highly polar compounds, such as substrates or products of CDA/DCTD, without the need for non-volatile ion-pairing reagents that can interfere, for example, with electrospray ionization. The high polarity of nucleotides, which limits their retention on reversed-phase columns, makes HILIC an attractive option. In a recent study by Huang et al. (2022), it was demonstrated that using an Acquity BEH amide column in HILIC mode enabled the complete separation of cytarabine monophosphate (ara-CMP), cytarabine diphosphate (ara-CDP), and cytarabine triphosphate (ara-CTP)—a task that could not be achieved using standard reversed-phase or ion-pair setups [67].

In addition to aminopropyl columns, zwitterionic stationary phase columns have shown promise due to their increased selectivity and stability over a wide range of pH conditions. Of these, the ZIC-HILIC (sulfoalkyl betaine on silica) and ZIC-cHILIC (phosphorylcholine on silica) columns have proved particularly effective. These columns maintain a fixed internal charge distribution (1:1 ratio of positive to negative charges), which is not affected by pH. As a result, the separation is mainly governed by hydrophilic partitioning combined with mild ionic interactions [68]. Ultimately, this transition from traditional ion-pair methods to modified HILIC-compatible mobile phases or hybrid approaches (e.g., reversed-phase chromatography with MS-friendly ion-pair reagents like hexylamine) represents a significant advancement in the analysis of nucleotide analogs.

Nonetheless, even HILIC presents its own challenges. While retention and baseline separation are improved, issues such as peak tailing and co-elution of endogenous nucleotides like CTP remain problematic in some matrices. These effects have been documented despite the use of high concentrations of ammonium acetate buffers. Additionally, as Crauste et al. (2009) previously observed, substantial concentrations of formic acid (up to 50 mM) still failed to efficiently elute triphosphorylated nucleotides from PGC columns, highlighting the challenge of balancing resolution and MS sensitivity [69].

### 3.2. LC-UV/Vis and LC-Fluorescence Based Assays

UV/Vis and fluorescence (FLD) detection are powerful spectrophotometric techniques whose fundamental principles, based on light absorption or emission by specific molecular groups, have been detailed in Chapter 2. When used alone, these techniques can be affected by interferences from compounds present in complex biological matrices. However, coupling them with LC significantly reduces this problem, as LC provides a preliminary separation of the sample components. This allows for higher selectivity and more accurate quantification of target analytes even in complex biological materials.

The following concise overview outlines the principles and advantages of combining LC with UV/Vis and FLD detection, including applications in the field of pyrimidine metabolism and the enzymatic activities of CDA and DCTD.

#### 3.2.1. LC-UV/Vis

The combination of LC with UV/Vis detection stands as one of the most commonly employed analytical techniques in bioanalysis for assessing CDA/DCTD enzymatic activity. This approach exploits the differential absorption of UV or Vis radiation by deaminase substrates (e.g., cytidine, deoxycytidine) and their corresponding products (e.g., uridine, deoxyuridine), as these molecules, being nucleotides, nucleosides, and purine/pyrimidine derivatives, naturally contain suitable chromophores (conjugated π-electron systems or aromatic structures). This enables the precise quantification of their conversion following chromatographic separation. Based on this principle, Ranjbarian et al. (2022) [58] distinguished substrates involved in the enzymatic reaction catalyzed by CDA/DCTD—such as cytidine and deoxycytidine—and their deamination products, uridine and deoxyuridine, based on their differential absorbance at 260 nm. LC-UV/Vis is also frequently employed in oncological pharmacology to measure drug concentrations or to study drug metabolism in plasma samples and other biological matrices [70].

#### 3.2.2. LC-FLD

For compounds that lack natural fluorescence (e.g., cytidine, uridine), chemical pretreatment—derivatization—is necessary using fluorescent reagents that selectively react with functional groups (e.g., hydroxyl, amino) to form strongly fluorescent products. A classical approach is pre-column derivatization with reagents such as 2-(5-chlorocarbonyl-2-oxazolyl)-5,6-methylenedioxybenzofuran (OMB-COCl), which enables fluorescent esterification of the sugar moiety of nucleosides. Nagaoka et al. (1992) [71] described the use of OMB-COCl for the simultaneous determination of ribonucleosides and 2′-deoxyribonucleosides, including cytidine and uridine, with detection limits in the range of 2–12 pmol.

To monitor the activity of the CDA and DCTD enzymes, samples containing cytidine or deoxycytidine are typically incubated with cell lysates or purified enzymes. Following derivatization, the LC-FLD method enables the detection of even small changes in the substrate/product ratio (cytidine/uridine or deoxycytidine/deoxyuridine). The resulting derivatives exhibit distinct retention times and fluorescence spectra, which facilitates their simultaneous quantification. This allows for sensitive and specific measurement of enzyme kinetics or inhibitory effects of tested compounds. These characteristics make LC-FLD a highly suitable method for in-depth studies of pyrimidine metabolism in both biochemistry and pharmacology.

### 3.3. LC-MS Based Assays

Over the past few years, LC-MS has become an outstanding technique for the quantitative analysis of drugs in the fields of pharmacokinetics and drug metabolism. Coupling LC with a mass spectrometer has significantly increased analytical sensitivity and specificity, enabling the detection of drugs and their metabolites at very low concentrations from minimal sample volumes. It also allows for the selective detection of analytes in complex matrices such as tissues or blood [70]. Thanks to these features, LC-MS has become the gold standard for determining the activity of CDA/DCTD.

MS enables the detection of molecules based on their mass-to-charge ratio (*m*/*z*), following their ionization in the gas phase. However, MS analysis of nucleosides and nucleotides—including substrates and products of CDA/DCTD such as cytidine/deoxycytidine and uridine/deoxyuridine—initially encountered several challenges. These included analytical issues such as the very low concentrations of target compounds in biological matrices, high polarity and low volatility of the analyzed molecules, their thermal lability, and the need for derivatization when using certain traditional methods. Such derivatization prolongs sample preparation, increases the risk of by-product formation, and may reduce both the yield and reproducibility of the analysis [72].

A major breakthrough in overcoming these issues was the development of so-called soft ionization techniques, particularly MALDI (Matrix-Assisted Laser Desorption/Ionization) and ESI (Electrospray Ionization), which made it possible to detect large, polar, and non-volatile biomolecules without the need for derivatization. In the context of nucleotide analysis, ESI is the most widely used ion source, offering gentle ionization conditions that are compatible with thermally labile and highly polar species such as mono-, di-, and triphosphorylated nucleotides. ESI facilitates the formation of multiply charged ions, thereby extending the accessible mass range and enabling the analysis of low-abundance species in complex matrices [72].

Tandem mass spectrometry (MS/MS), often operated in multiple reaction monitoring (MRM) mode on triple quadrupole instruments, provides additional specificity by isolating precursor ions and analyzing their characteristic fragmentation patterns [73]. This is particularly advantageous for the identification of isobaric nucleotide species or for differentiating closely related structural analogs, including epigenetically modified or chemically altered nucleotides. Depending on the ionization mode and collision energy settings, fragmentation typically proceeds via the loss of phosphate moieties, nucleobase-specific cleavages, or sugar-phosphate backbone dissociation.

The combination of MS/MS with ESI has proven to be an exceptionally powerful tool for determining CDA/DCTD activity, as well as for the quantification and monitoring of biologically active nucleoside and nucleotide derivatives. For example, Zhou et al. (2018) [74] developed and validated an LC-ESI-MS/MS method to clarify the in vivo exposure of the compound FY363 and its metabolite gemcitabine following FY363 administration. This method, among other things, indirectly monitors CDA/DCTD activity by measuring the levels of gemcitabine metabolites. In contrast, the study by Huang et al. (2022) focused on the stability of cytarabine and its metabolites ara-CMP, ara-CDP, and ara-CTP in the HL-60 cancer cell line, as influenced by CDA/DCTD activity. For this purpose, they developed an LC-ESI-MS/MS method that can quantify not only cytarabine itself but also the phosphorylated metabolites ara-CMP, ara-CDP, and ara-CTP [67]. Similarly to gemcitabine, cytarabine, or other cytidine analogs, the efficacy of 5-azacytidine in the treatment of MDS or AML can be compromised by the activity of the enzyme CDA. Therefore, Donnette et al. (2023) later developed a rapid LC-MS/MS method for the quantification of 5-azacytidine in human plasma [75].

#### Measurement of Enzyme Activity Using LC-MS

**Figure 9:**
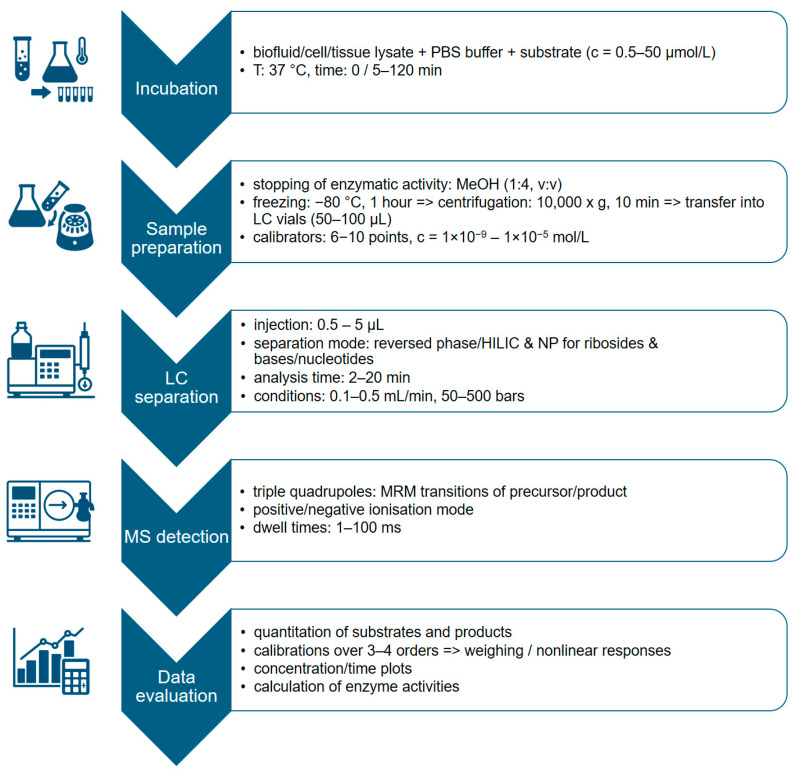
Workflow for measuring enzyme activity by LC-MS.

LC, especially HPLC, is one of the techniques used to determine the enzymatic activity of CDA and DCTD. Modern approaches combine LC with mass spectrometry (LC-MS or LC-MS/MS), allowing not only the quantification of substrates and products, but also enzyme inhibitors such as tetrahydrouridine (THU), directly in biological matrices [76,77]. These methods are significantly more sensitive than traditional spectrophotometric or radioactive methods, and provide accurate results without the need for substrate labeling or synthetic modifications that could affect the kinetics of enzymatic reactions [78]. The schematic workflow for measuring enzyme activity using LC-MS is shown in Figure 9.

The analysis of substrates and products associated with CDA and DCTD activity primarily utilizes HPLC- or LC-MS/MS techniques for the quantitative monitoring of substrate conversion to the corresponding uridine products. For example, Ligasova et al. (2023) employed LC-MS to track the catabolic conversion of cytidine and deoxycytidine to uridine and deoxyuridine, which were analyzed in cell lysate samples from nine different cell lines, including cancer cell lines, diploid cell lines, and an immortalized diploid cell line [21].

However, optimization of chromatographic parameters is essential for the effective separation of substrates and products in enzymatic reactions. Critical factors include the composition of the mobile phase, the selection of a suitable column type, and the choice of appropriate detection methods such as fluorescence, UV spectroscopy, or LC-MS [79], where isotopically labeled internal standards (e.g., ^13^C-, ^2^H-, or ^15^N-labeled substrates) are frequently employed to ensure accurate quantification and to account for matrix effects and instrument variability. For example, Krijt et al. (2009) used ^2^H_3_- and ^13^C_5_-labeled internal standards in LC-MS/MS analysis of tissue extracts to achieve reliable quantification [80]. Special attention must be given to the concentration of the mobile phase and pH, as suboptimal conditions can significantly compromise the quality of separation. Inadequate pH or ionic strength may result in poor resolution, overlapping peaks, or unpredictable shifts in retention times, thereby affecting the accuracy and reproducibility of analytical results [81]. 

CDA activity is mainly measured in the context of the conversion of cytidine analogs used in medicine as anticancer drugs. An example is the determination of CDA activity in the inactivation of gemcitabine (2′,2′-difluoro-2′-deoxycytidine, dFdC) or its analogs by LC-MS/MS [74]. Gemcitabine requires intracellular phosphorylation to the cytotoxic form—2′,2′-difluoro-2′-deoxycytidine triphosphate (dFdCTP), which incorporates into DNA and causes the termination of DNA chain replication [82]. However, gemcitabine can be deaminated to its therapeutically inactive uridine counterpart, difluorodeoxyuridine (dFdU), suggesting that tumors with high CDA expression may be resistant to treatment involving the administration of cytidine analogs such as gemcitabine. Similarly to gemcitabine, which can be deaminated by CDA, its monophosphate form, 2′,2′-difluorodeoxycytidine 5′-monophosphate (dFdCMP), can be deaminated by DCTD to form 2′,2′-difluoro-2′-deoxyuridine monophosphate (dFdUMP) [83]. CDA activity can contribute to treatment resistance even in the case of nucleoside analogs used for the treatment of hematological malignancies, such as decitabine and 5-azacytidine, which are catabolized to their uridine counterparts—therapeutically inactive products [84]. In contrast, Abarra and colleagues studied the stability of cytarabine in the treatment of patients with AML. Using LC-MS/MS, they found that cytarabine was degraded by CDA activity, but that addition of THU, a CDA inhibitor, stabilized cytarabine in patient plasma samples [85]. As with previous nucleoside analogs, cytarabine is subject to undesirable deamination by the action of CDA to its uridine product, uracil arabinoside. Similarly, the monophosphate form of ara-CMP may be subject to degradation by DCTD activity to ara-UMP, the therapeutically inactive product [24,86]. 

### 3.4. Summary and Comparison of Liquid Chromatography-Based Methods for CDA and DCTD Activity Analysis

Chromatography-based methods play a key role in the determination of CDA and DCTD enzymatic activities, especially when studying pyrimidine metabolism and the impact of cytostatic agents. The use of different chromatographic approaches enables the effective separation of structurally similar nucleotides, such as dCMP and CMP, which are difficult to distinguish using conventional techniques [64]. LC-UV/Vis is commonly used to monitor CDA and DCTD deamination reactions based on absorbance differences between substrates and products (e.g., cytidine vs. uridine) at specific wavelengths. A major advantage of combining UV/Vis detection with chromatographic separation is the increased selectivity, which allows for effective elimination of interferences from complex biological matrices. Furthermore, derivatization of the target molecules with fluorescent or other tags is unnecessary, thereby simplifying both sample preparation and the overall analysis process.

In contrast, LC-FLD detection requires derivatization of non-fluorescent compounds using reagents such as OMB-COCl and allows for highly sensitive monitoring of nucleosides, including cytidine and uridine, down to the picomole level [71]. LC-FLD offers key advantages such as high sensitivity, selectivity, and low background noise. The highest sensitivity and specificity, however, is achieved with LC-MS, which is considered the gold standard for both quantitative and qualitative assessment of CDA/DCTD activity as well as for the general analysis of metabolites in biological matrices. LC-MS also allows for the simultaneous detection of therapeutic inhibitors, such as THU, without the need for labeling or derivatization. Nevertheless, it is important to consider that this method is susceptible to the matrix effect, where components of the biological sample interfere with ionization efficiency [70].

ESI, which is commonly used in LC-MS, enables the soft ionization of non-volatile and polar molecules such as nucleotides without degrading them. The combination of chromatography with MS/MS and MRM ensures high detection specificity even in the presence of interfering substances. Thus, LC-MS proves to be an indispensable tool not only for biochemical research on CDA and DCTD, but also for pharmacological and clinical applications focused on the metabolism and efficacy of cytidine analogs.

## 4. Radiometric Assays

Radiometric enzyme assays are techniques used to determine the rate of an enzymatic reaction by measuring the radioactivity in the product or the remaining substrate [87]. It is based on the use of radioactively labeled substrates, typically nucleosides or nucleotides tagged with isotopes such as ^3^H or ^14^C, which are converted by the enzyme to labeled products. These reaction products are then separated by chromatographic techniques, such as thin-layer chromatography, paper chromatography, or ion-exchange chromatography. Following this, the radioactivity is quantified using liquid scintillation counting to provide a sensitive measure of enzymatic activity [88,89,90].

This approach offers several advantages, notably its sensitivity, which allows for the detection of enzymatic activity even at very low concentrations. It is especially valuable when colorimetric or fluorometric assays lack the required detection limits. However, disadvantages include concerns related to radiation safety, the need for specialized facilities and handling protocols, and the generation of radioactive waste, which necessitates appropriate disposal procedures in compliance with regulatory guidelines.

Several studies utilizing radioisotopically labeled substrates have demonstrated the effectiveness of radiometric assays in investigating CDA activity and cytarabine metabolism in hematologic cells. For instance, radiotracer-based assays were employed to assess CDA activity in both normal and leukemic granulocytes, using [^14^C]-cytidine, [^3^H]-cytarabine, and [^14^C]-deoxycytidine as substrates. Partially purified CDA from normal human granulocytes exhibited higher affinity for its physiological substrate cytidine (K_m_ = 11 µM) than for the chemotherapeutic analog cytarabine (K_m_ = 88 µM). K_m_ (Michaelis constant) is a measure of the enzyme’s affinity for the substrate; a low Kₘ value indicates high affinity. In contrast to normal granulocytes, leukemic cells demonstrated a significant reduction in CDA concentration, which was strongly correlated with cellular immaturity [91]. Pérignon and colleagues (1985) characterized CDA activity in peripheral blood mononuclear cells using a radioisotopic assay based on the enzymatic conversion of [2-^14^C]-cytidine and [U-^14^C]-2-deoxycytidine to their deaminated products. Their results showed significant reduction in CDA activity in lymphoblasts from patients with acute lymphoblastic leukemia (ALL), with a correlation between enzymatic activity and the percentage of circulating blasts [90]. In a comprehensive study by Chou et al. (1977), a radiometric assay was employed to evaluate the intracellular metabolism of cytarabine in leukemic and normal hematopoietic cells. The researchers used [^3^H]-cytarabine to trace its conversion to the cytarabine-CTP, which was quantified following separation by ion-exchange chromatography. Radioactivity in the nucleotide fractions was measured via liquid scintillation counting, enabling the assessment of cytarabine-CTP synthesis in different leukemic subtypes. The assay revealed significantly higher cytarabine-CTP accumulation in previously untreated AML blasts from patients who achieved complete remission, compared to those with chronic lymphocytic leukemia, acute lymphoblastic leukemia, and healthy donors [92].

Similarly to CDA, DCTD has been studied using radiolabeled substrates. In a study by Giusti et al. (1970), [2-^14^C]-dCMP was used to determine DCTD activity in normal and malignant human tissues. After enzymatic conversion, the radiolabeled product dUMP was isolated by thin-layer chromatography on DEAE-cellulose, and subsequently quantified using radiometric detection [93]. Maley and Maley (1964) similarly utilized dCMP-2-^14^C in kinetic studies of purified dCMPase from chick embryos, using ion-exchange chromatography to isolate the radiolabeled product. Their findings demonstrated a strong regulatory effect of dCTP (activation) and dTTP (noncompetitive inhibition), supporting an allosteric mechanism of control [94]. In microbial systems, radiometric assays with [^32^P]-dCMP were used to characterize bacteriophage T4-induced dCMPase in *E. coli*, further validating the utility of the technique across biological models [95]. Extending these applications to clinical hematology, Ellims and Medley (1984) measured dCMPase activity in lymphoid malignancies using [U-^14^C]dCMP, and found that enzyme activity was elevated, particularly in more aggressive forms of acute lymphoblastic leukemia and high-grade non-Hodgkin lymphomas [96]. Despite considerable variability within individual disease subtypes, the findings suggest that dCMPase activity may serve as a biochemical marker of tumor aggressiveness.

In conclusion, although radiometric enzyme assays were historically essential due to their high sensitivity, they are increasingly being used less frequently today. Handling radioactive materials requires strict safety protocols, specialized equipment, and produces hazardous waste. With the development of non-radioactive techniques such as mass spectrometry and fluorescence-based assays, radiometric methods are increasingly being replaced. Still, in specific cases requiring ultra-sensitive detection, they remain valuable.

## 5. Cell-Based Assays

Cell-based methods allow us to evaluate the activity of CDA and DCTD directly within intact cells. These are relatively new strategies, and their development and broader application are closely tied to significant advancements in digital image processing and analysis. Modern microscopic systems and sophisticated software tools now enable automated analysis of large sets of image data, opening the door for quantitative evaluation of cell-based assays in a high-throughput format. Cell-based assays rely on the ability of cells to transport, metabolize, and subsequently incorporate modified nucleoside analogs into DNA or RNA. Key substrates in cell-based assays include (deoxy)cytidine analogs such as 5-ethynyl-2′-deoxycytidine (EdC) [21] or 5-fluorocytidine (FC) [1]. After their deamination, the corresponding uridine analogs (EdU; 5-ethynyl-2ʹ-deoxyuridine, or its monophosphate, or 5-fluorouridine) are formed. These are then further phosphorylated and incorporated into nucleic acids. The detection of incorporated products serves as an indirect indicator of enzymatic activity within the cellular context. These approaches often use indirect visualization of deamination products or monitor the cellular response to the enzymes’ substrates.

The history of EdC use is quite interesting. Originally, this analog, much like EdU [97], was primarily considered and even commercially marketed as a DNA replication marker. Its supposed advantage over EdU was its lower cytotoxicity [98]. However, a later detailed study by Ligasova et al. (2016) revealed that EdC is hardly incorporated into DNA, or only in negligible amounts [99]. Instead, EdC is enzymatically deaminated to EdU within cells, and it is EdU (after phosphorylation to EdUTP; 5-ethynyl-2ʹ-deoxyuridine triphosphate) that is subsequently incorporated into DNA. The observed lower toxicity of EdC compared to EdU was not due to its inherent harmlessness upon incorporation, but rather because only a portion of EdC was typically converted to the more toxic EdU [99]. These findings paved the way for using EdC specifically as a tool to study deaminase activity. It was shown that EdC is metabolized via two main deamination pathways. The first pathway involves the direct deamination of EdC to EdU by CDA. The second pathway first involves the phosphorylation of EdC by dCK to 5-ethynyl-2′-deoxycytidine monophosphate (EdCMP), which can then be deaminated by DCTD to 5-ethynyl-2′-deoxyuridine monophosphate (EdUMP) [21,99]. The resulting EdU, or EdUMP (further phosphorylated to EdUTP), is then incorporated into newly synthesized DNA during replication. The incorporated EdU is subsequently detected using a Cu(I)-catalyzed cycloaddition reaction (“click” chemistry) with a fluorescently labeled azide. This method does not require DNA denaturation, which is a significant advantage over traditional BrdU (5-bromo-2ʹ-deoxyuridine) detection [97]. The work by Ligasova et al. (2023) also demonstrated that although EdC is a substrate for CDA, it is deaminated with lower efficiency than native cytidine [21].

To distinguish the contributions of CDA and DCTD to EdU production from EdC, THU, a specific CDA inhibitor, is used. Incubating cells with EdC in the presence and absence of THU allows for an estimation of CDA’s relative contribution to the EdC to EdU conversion. A significant reduction in EdU incorporation after THU addition indicates a dominant role for CDA. Conversely, a minor effect of THU suggests low or absent CDA activity, with the remaining conversion likely occurring via DCTD. This approach, thus, enables the rapid identification of cell lines with a functional CDA deficiency. It is also useful for identifying lines with low or absent DCTD activity. If EdU incorporation does not occur after CDA inhibition with THU, low or absent DCTD activity is indicated. Another option is to use cell lines with genetically induced CDA or DCTD deficiencies (e.g., via siRNA) to study the specific role of the second enzyme [21].

The advantages of these EdC/EdU-based methods include rapid detection using “click” chemistry, the ability to use them on fixed cells and lysates, and their compatibility with fluorescence microscopy or microplate readers. They also allow for simultaneous testing of cytotoxicity and replication activity. The main disadvantage is that the amount of incorporated EdU results from the interplay of multiple cellular processes. Additionally, THU can exhibit CDA-independent antiproliferative effects, which must be considered. Method variants can also use other cytidine analogs, such as halogenated ones, though their detection typically requires antibodies and DNA denaturation [99].

Another cell-based approach involves the immunocytochemical detection of 5-fluorouridine (FU) incorporated into cellular RNA after administering FC [1]. In cells with active CDA, FC is deaminated to FU, which is then phosphorylated and incorporated into RNA. This conversion and FU incorporation into RNA do not occur in CDA-deficient cells. FU detection in RNA is performed using a primary antibody against BrdU, which cross-reacts with FU, and a fluorescently labeled secondary antibody. This method allows for the identification of cells with functional/non-functional CDA in about two hours and does not require DCTD inhibition. Its main advantages are speed, simplicity, and the ability of visualization at the single-cell level. A study also showed a correlation between the FU/FC cytotoxicity ratio and CDA content, suggesting prognostic potential. A disadvantage can be the need for antibodies. Moreover, while the method is excellent for identifying functionally deficient cells, it has a limited ability to precisely quantify different levels of CDA activity among proficient lines. This was confirmed by the authors of the method, who stated that their analysis could not establish a direct correlation between the fluorescence intensity and the amount of the CDA enzyme in various cell lines [1].

In conclusion, cell-based assays relying on the incorporation of modified nucleoside analogs, such as EdC or FC, and subsequent detection of their metabolites, offer relatively high sensitivity. This sensitivity primarily stems from the detection occurring directly within cells, where specific signals (e.g., fluorescence of incorporated EdU or FU) are well-differentiated from background, minimizing interference from extracellular substances or lysate components that can complicate biochemical assays. Despite this advantage, these methods are more suited as qualitative or semi-quantitative indicators of the presence or absence of functional deaminase activity (e.g., for rapid identification of CDA-deficient lines), or for comparing relative differences between cell lines under defined conditions. For precise quantification of enzymatic activity (e.g., determining specific enzyme activity), methods performed on cell lysates or with purified enzymes are still more appropriate, as substrate and enzyme concentrations and reaction time can be better controlled. However, cell-based assays are invaluable for studying enzyme function in their natural physiological context.

## 6. Considerations for Method Selection and Application

The selection of an appropriate assay is a critical step, guided by the specific research question, available resources, and the nature of the desired data. A fundamental distinction lies between endpoint assays, which measure the total product accumulated over a set period, and real-time kinetic assays, which monitor the initial reaction rate. While endpoint methods can be made highly sensitive for low-activity samples through prolonged incubation, kinetic assays provide more biochemically accurate data.

For rapid kinetic analysis of purified enzymes, direct spectrophotometry is a straightforward choice. When high-throughput screening is the objective, plate-based indirect assays or direct fluorogenic assays are most suitable. For analyses requiring the highest accuracy and specificity in complex biological matrices like plasma, LC-MS is the undisputed gold standard. Finally, to investigate enzyme function within a physiological context, cell-based assays are invaluable, offering insights that in vitro methods cannot.

To further aid in this selection, Table 1 provides a comparative overview of the discussed methodologies, including columns for ‘Sensitivity’ and ‘Feasibility/Cost’. The ‘Sensitivity’ values are presented as an estimated Limit of Detection (LOD) based on the literature discussed in this review. It is important to note that for indirect assays, this value refers to the detection limit for the reporter molecule (e.g., ammonia), while for direct assays, it refers to the substrate. For cell-based assays, sensitivity is described in a biological context rather than as a molar concentration, reflecting the unique advantages of these techniques. The ‘Feasibility/Cost’ column provides a general ranking based on typical requirements for equipment, reagents, and specialized facilities.

## 7. Concluding Remarks and Future Prospects

The accurate measurement of CDA and DCTD activity is crucial for both fundamental research into pyrimidine metabolism and for clinical applications related to the efficacy of nucleoside analog chemotherapeutics. As this review has outlined, researchers have a diverse toolkit at their disposal, ranging from classic spectrophotometric assays to highly advanced mass spectrometry and cell-based approaches. The selection of an appropriate method is not trivial and must be carefully tailored to the specific research question, considering factors such as required sensitivity, sample complexity, available equipment, and whether the goal is quantitative analysis or a more qualitative assessment of enzyme function within a physiological context.

The current trend is clearly moving towards methods that offer maximum sensitivity and specificity. LC-MS has firmly established itself as the gold standard for the precise quantification of substrates, products, and inhibitors, especially in complex biological matrices [70,72,76]. At the same time, cell-based assays [1,21] are gaining prominence as invaluable tools for studying enzyme activity in a native cellular environment, providing insights that cannot be obtained from lysate-based assays alone. While these methods are often semi-quantitative, their ability to reflect the interplay of transport, metabolism, and incorporation makes them uniquely powerful.

Looking forward, several exciting avenues are poised to further advance the field. The development of more robust and cost-effective direct fluorogenic substrates [53,54] will be critical for enabling high-throughput screening of large compound libraries to discover novel deaminase inhibitors. Furthermore, the frontier of single-cell analysis presents a significant opportunity. Adapting fluorescence-based methods to quantify deaminase activity at the single-cell level could reveal crucial information about cellular heterogeneity within tumors, potentially explaining why some cancer cells survive therapy while others do not.

## Figures and Tables

**Figure 5:**
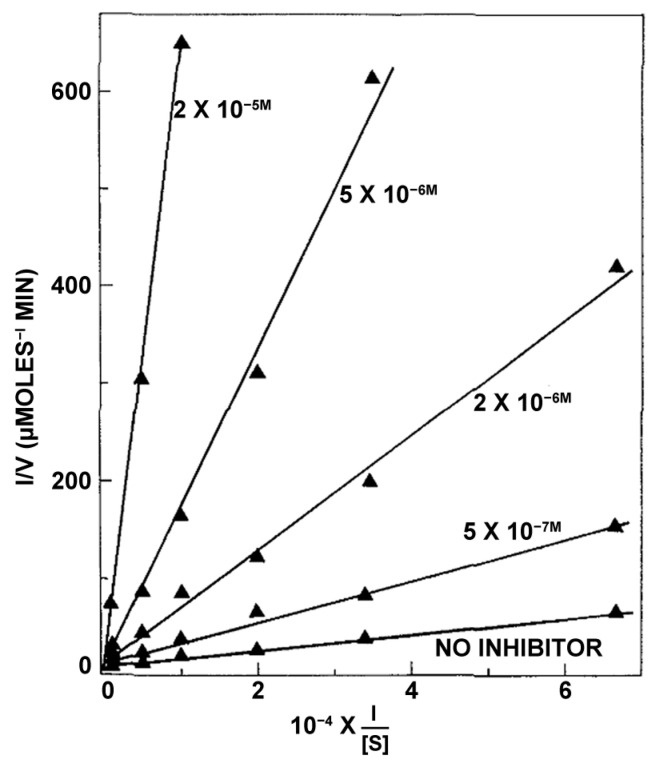
Example of the measurement of deaminase activity using direct UV-Vis spectrophotometric assay. The graph represents reciprocal plot of the rate of deamination of cytidine at 25 °C, in 0.05 M Tris-HCl buffer, pH 7.5, with 0.05 unit per ml of deaminase in the presence of varying concentrations of 3,4,5,6-tetrahydrouridine. Adapted from [43].

**Table 1:** Methods overview and comparison.

Method Type	Advantages	Disadvantages	Ref.	Sensitivity (LOD)	Feasibility/Cost	Assay Type
Spectrophotometric						
Direct	Simple, rapid, cost-effective, real-time, non-destructive.	Sensitivity to other UV-absorbers, requires pure samples, light scattering issues, limited sensitivity.	[43,44]	LOD: ~1–10 µM (e.g., for cytidine/uridine)	Low	Kinetic
Indirect	High sensitivity, complex sample applicability, high-throughput adaptable.	Endogenous ammonia/metabolic interference, high background, long protocols, enzyme coupling optimization needed.	[46,47,48,49]	LOD: ~50 µM (for ammonia)	Low	Endpoint
Fluorimetric						
Direct	High sensitivity, real-time monitoring, suited for kinetics/inhibitor screening, red-shifted spectra.	Complex/costly substrate synthesis, kinetic parameters may differ from native substrates.	[53,54]	LOD: ~0.1–1 µM (for fluorescent analog)	Medium-High	Kinetic
Indirect	Very high sensitivity (picomolar to subpicomolar detection limits for ammonia).	Endogenous ammonia interference, limited dynamic range.	[50,51]	LOD: ~10–100 nM (for ammonia)	Low-Medium	Endpoint
Liquid Chromatography-Based Assays						
	High resolution and selectivity, suitable for complex samples, adaptable to various detection modes.	Time-consuming method optimization, high reagent/equipment cost.				
LC-UV/Vis	No derivatization, simpler sample preparation.	Sample clarity required, limited sensitivity.	[58]	LOD: ~0.1–10 µM (e.g., for cytidine/uridine, deoxycytidine/deoxyuridine)	Medium	Endpoint
LC-FLD	High sensitivity (picomole level), high selectivity, low background noise.	Requires derivatization, sensitive to fluorescence quenching/autofluorescence.	[71]	LOD: ~0.1–1 µM (e.g., for cytidine/uridine derivatized by fluorescent reagents)	Medium-High	Endpoint
LC-MS	Highest sensitivity and specificity, no derivatization, complex matrix applicability, simultaneous inhibitor detection.	High cost of equipment, time-consuming method development/sample preparation, susceptible to matrix effect (interference with ionization efficiency).	[70,72]	LOD: ~1–100 nM (e.g., for cytidine/uridine, deoxycytidine/deoxyuridine)	High	Endpoint
Radiometric Assays						
Radiometric Assays	High sensitivity for low concentrations of enzymes.	Radiation safety concerns, specialized facilities/protocols, hazardous waste generation, declining use.	[91,100]	LOD: <1 nM; enables detection of low analyte levels in complex mixtures	Medium-High	Endpoint
Cell-Based Assays						
	Physiological context study, compatible with fluorescence microscopy/microplate readers, high sensitivity.	Product amount reflects multiple cellular processes (transport, metabolism), limiting precise quantification.		High (biological context); ability to detect activity at the single-cell level	Medium	Endpoint
EdC-to-EdU conversion assay	Rapid detection (“click” chemistry), no DNA denaturation, simultaneous cytotoxicity/replication testing.	Replication-dependent (limits analysis to proliferating cells).	[21]			
FC-to-FU conversion assay	Transcription-dependent (broader applicability across cell cycle phases/quiescent cells).	Antibody use.	[1]			

## Data Availability

No new data were created or analyzed in this study. Data sharing is not applicable to this article.

## Abbreviations

The following abbreviations are used in this manuscript:

CDA	Cytidine deaminase
DCTD	Deoxycytidine monophosphate deaminase; dCMP deaminase
dCTP	2′-Deoxycytidine 5′-triphosphate
ATP	Adenosine 5′-triphosphate
dCMP	2′-Deoxycytidine 5′-monophosphate
dUMP	2′-Deoxyuridine 5′-monophosphate
dTTP	Thymidine 5′-triphosphate
hmdCMP	5-Hydroxymethyl-2′-deoxycytidine 5′-monophosphate
hmdUMP	5-Hydroxymethyl-2′-deoxyuridine 5′-monophosphate
dNTP	Deoxynucleotide triphosphate
MDS	Myelodysplastic syndrome
AML	Acute myeloid leukemia
dCa	2′-Deoxycytidine analog
dUa	2′-Deoxyuridine analog
dCaMP	Monophosphate form of dCa
dCaDP	Diphosphate form of dCa
dCaTP	Triphosphate form of dCa
dCK	Deoxycytidine kinase
UCK	UMP-CMP kinase
NDK	Nucleoside diphosphate kinase;
5′-NT	5′-Nucleotidases
dUaMP	Monophosphate form of dUa
FdUMP	5-Fluoro-2′-deoxyuridine monophosphate
5hmdC	5-Hydroxymethyl-2ʹ-deoxycytidine
5fdC	5-Formyl-2ʹ-deoxycytidine
5hmdU	5-Hydroxymethyl-2ʹ-deoxyuridine
5fdU	5-Formyl-2ʹ-deoxyuridine
SNP	Single nucleotide polymorphism
UV	Ultraviolet
Vis	Visible
GLDH	Glutamate dehydrogenase
NADH	Nicotinamide adenine dinucleotide
OPA	o-Phthaldialdehyde
EdC	5-Ethynyl-2ʹ-deoxycytidine
FC	5-Fluorocytidine
EdU	5-Ethynyl-2ʹ-deoxyuridine
FU	5-Fluorouridine
EdUTP	5-Ethynyl-2ʹ-deoxyuridine triphosphate
EdCMP	5-Ethynyl-2ʹ-deoxycytidine monophosphate
EdUMP	5-Ethynyl-2ʹ-deoxyuridine monophosphate
BrdU	5-Bromo-2ʹ-deoxyuridine
THU	Tetrahydrouridine
HPLC	High-performance liquid chromatography
RP-LC	Reversed-phase liquid chromatography
NP-LC	Normal-phase liquid chromatography
HILIC	Hydrophilic interaction chromatography
OMB-COCl	2-(5-Chlorocarbonyl-2-oxazolyl)-5,6-methylenedioxybenzofuran
LC-MS/MS	Liquid chromatography with tandem mass spectrometry
rNTP	Ribonucleotide triphosphate
ADP	Adenosine 5′-diphosphate
PGC	Porous graphitic carbon column
CMP	Cytidine 5′-monophosphate
CTP	Cytidine 5′-triphosphate
dFdC	2′,2′-Difluoro-2′-deoxycytidine
dFdCTP	Gemcitabine triphosphate (2′,2′-Difluorodeoxycytidine 5′-triphosphate)
dFdU	2′,2′-Difluoro-2′-deoxyuridine
dFdCMP	Gemcitabine monophosphate (2′,2′-Difluorodeoxycytidine 5′-monophosphate)
ara-CMP	Cytarabine monophosphate
ara-CDP	Cytarabine diphosphate
ara-CTP	Cytarabine triphosphate
MALDI	Matrix-assisted laser desorption/ionization
ESI	Electrospray ionization
LB medium	The Lysogeny Broth medium
M9 medium	M9 minimal salts medium
tzC	Isothiazolo[4,3-d]pyrimidine analog of cytidine
thC	Thieno[3,4-d]pyrimidine analog of cytidine
mthC	Methylthieno[3,4-d]pyrimidine analog of cytidine
UV-Vis	Ultraviolet-Visible
TS	Thymidylate synthase
LC	Liquid chromatography
MS	Mass spectrometry
FLD	Fluorescence detection
MS/MS	Tandem mass spectrometry
dFdUMP	2′,2′-Difluoro-2′-deoxyuridine monophosphate
Cytarabine	Ara-C; 1-β-D-arabinofuranosylcytosine
MRM	Multiple reaction monitoring
LOD	Limit of detection
ALL	Acute lymphoblastic leukemia
